# Alleviation of LPS-Induced Inflammation and Septic Shock by *Lactiplantibacillus plantarum* K8 Lysates

**DOI:** 10.3390/ijms22115921

**Published:** 2021-05-31

**Authors:** Gayoung Kim, Kyeong-Hun Choi, Hangeun Kim, Dae-Kyun Chung

**Affiliations:** 1 Graduate School of Biotechnology, Kyung Hee University, Yongin 17104, Korea; gayoung95@gmail.com (G.K.); chlkh6599@naver.com (K.-H.C.); 2 Research and Development Center, Skin Biotechnology Center Inc., Yongin 17104, Korea; 3 Skin Biotechnology Center, Kyung Hee University, Suwon 16229, Korea

**Keywords:** *Lactiplantibacillus plantarum* K8, probiotic lysates, THP-1, inflammatory cytokines, TNF-α, IL-1β, lipopolysaccharide (LPS), septic shock

## Abstract

We previously showed that *Lactiplantibacillus plantarum* K8 and its cell wall components have immunoregulatory effects. In this study, we demonstrate that pre-treatment of *L. plantarum* K8 lysates reduced LPS-induced TNF-α production in THP-1 cells by down-regulating the early signals of mitogen-activated protein kinase (MAPK) and nuclear factor-κB (NF-κB). The down-regulation of signals may be caused by the induction of negative regulators involved in toll-like receptor (TLR)-mediated signaling. However, co-treatment with high concentrations of *L. plantarum* K8 lysates and lipopolysaccharide (LPS) activated the late signaling of extracellular signal-regulated kinase (ERK), c-Jun N-terminal kinase (JNK), and NF-κB pathways and resulted in the induction of absent in melanoma 2 (AIM2) inflammasome-mediated interleukin (IL)-1β secretion. Intraperitoneal injection of *L. plantarum* K8 lysates in LPS-induced endotoxin shock mice alleviated mortality and reduced serum tumor-necrosis factor (TNF)-α, IL-1β, aspartate aminotransferase (AST) and alanine aminotransferase (ALT) levels. In addition, the mRNA levels of TNF-α, IL-1β, and IL-6 decreased in livers from mice injected with *L. plantarum* K8 followed by LPS. Hematoxylin and eosin (H&E) staining of the liver showed that the cell size was enlarged by LPS injection and slightly reduced by *L. plantarum* K8 lysate pre-injection followed by LPS injection. Macrophage infiltration of the liver also decreased in response to the combination injection compared with mice injected with only LPS. Taken together, our results show that although *L. plantarum* K8 lysates differentially regulated the production of LPS-induced inflammatory cytokines in THP-1 cells, the lysates inhibited overall inflammation in mice. Thus, this study suggests that *L. plantarum* K8 lysates could be developed as a substance that modulates immune homeostasis by regulating inflammation.

## 1. Introduction

Inflammation is one of the biological responses to stimuli and is a protective response that involves immune cells, blood vessels, and inflammatory mediators. Acute inflammation inhibits cellular damage in the early stages, removes damaged tissue and necrotic cells from the wound, and usually plays a role in healing [1]. However, inflammation is a double-edged sword. Too little inflammation can cause the gradual destruction of tissues by harmful stimuli (such as bacteria) and impair the survival of the organism [2]. In contrast, chronic inflammation can cause various diseases, such as allergies, cancer, and arthritis, and in severe cases it can lead to sepsis [3]. Currently, various anti-inflammatory drugs, such as non-steroidal anti-inflammatory drugs, are used to treat inflammatory diseases, but these drugs are generally associated with infections and side effects including gastrointestinal and cardiovascular adversities [4,5].

The inflammatory response is mediated through a variety of cytokines. Tumor necrosis factor (TNF)-α promotes the inflammatory response and is produced primarily by activated macrophages [6]. Although TNF-α plays an important role in the host defense against pathogen invasion [7], dysregulation of TNF-α production has been implicated in a variety of human diseases including Alzheimer’s disease, cancer, major depression and inflammatory bowel disease (IBD) [8,9,10,11]. Anti-TNF-α biologics are used to suppress inflammation, but several side effects have been reported, such as aggravated disease and effects on the nervous system and autoimmunity [12,13]. Interleukin (IL)-1β is produced as a proprotein by activated macrophages and is proteolytically processed by caspase-1 into its active form [14]. IL-1β is also an important mediator of the inflammatory response and is involved in a variety of cellular activities, including cell proliferation, differentiation, and apoptosis [15]. Increased production of IL-1β leads to a variety of auto-inflammatory syndromes [15].

Due to the above-mentioned side effects of anti-inflammatory drugs, many studies are being conducted to investigate the suppression of inflammation using probiotics. For example, it has been reported that many strains of *Lactiplantibacillus plantarum* (*L. plantarum*) have anti-inflammatory effects [16,17]. This anti-inflammatory effect of *L. plantarum* is induced through the interaction of cell wall components such as lipoteichoic acid (LTA)and lipoprotein, with toll-like receptor (TLR) 2 in host cells [18,19,20]. We have confirmed that LTA, one of the cell wall components of *L. plantarum* K8, has been shown to have a strong immunomodulatory ability over the past 20 years. In particular, LTA of *L. plantarum* K8 alleviates septic shock induced by lipopolysaccharide (LPS) [21], inhibits C3 production induced by cytokines [22], down-regulates matrix metalloproteinase (MMP)-1 production induced by ultraviolet (UV) [23], and suppresses lipopolysaccharide (LPS)-mediated atherosclerotic plaque inflammation [24], indicating that LTA of *L. plantarum* K8 has a great effect in alleviating inflammation-related diseases. However, the efficacy of *L. plantarum* lysate on inflammation control has not yet been studied. In this study, we investigated whether *L. plantarum* K8 lysates regulated cytokines in immune cells. The expressions of cytokines and other inflammation-related biomarkers were also examined in endotoxin shock mice.

## 2. Results

### 2.1. Cell Viability Assay of THP-1 Cells Treated with L. plantarum K8 Lysate and LPS

**Figure 1:**
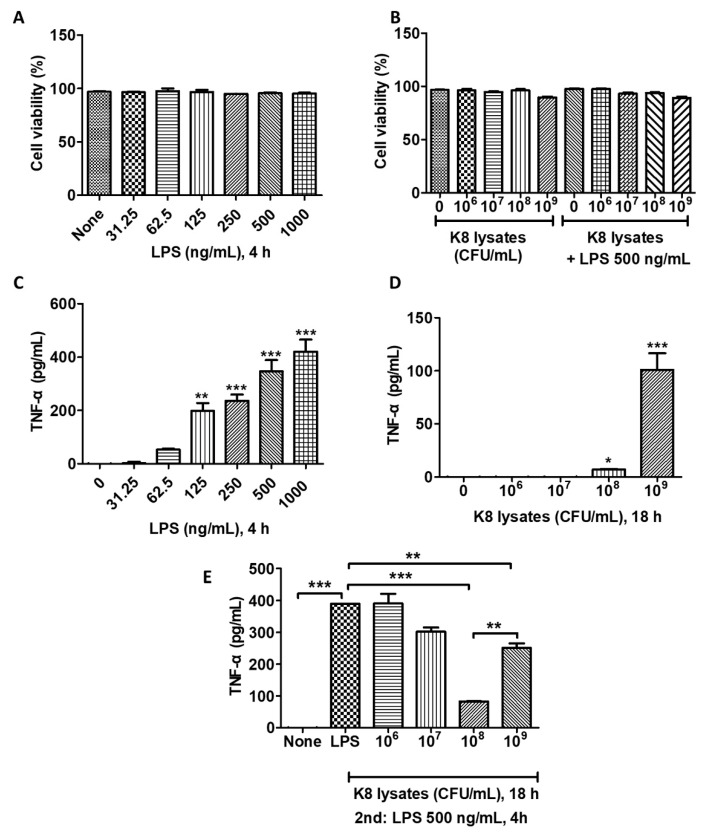
*L. plantarum* K8 lysates inhibited lipopolysaccharide (LPS)-induced tumor necrosis factor (TNF)-α production in THP-1 cells. (**A**) THP-1 cells were treated with LPS at the indicated concentrations for 4 h. (**B**) THP-1 cells were pretreated with *L. plantarum* K8 lysates at the indicated concentrations for 18 h and treated with 500 ng/mL LPS for 4 h. In (**A**,**B**), cell viability was measured using Trypan blue staining method. (**C**) THP-1 cells were treated with LPS at the indicated concentrations for 4 h. (**D**) THP-1 cells were treated with *L. plantarum* K8 lysates from 1 × 10^6^ to 1 × 10^9^ colony forming unit (CFU)/mL for 18 h. (**E**) THP-1 cells were pretreated with *L. plantarum* K8 lysates at the indicated concentrations for 18 h and treated with 500 ng/mL LPS for 4 h. In (C-E), the TNF-α level in the culture supernatants was determined using enzyme-linked immunosorbent assay (ELISA). Statistical analysis was conducted with one-way ANOVA followed by Tukey’s HSD post hoc test. * *p* < 0.05; ** *p* < 0.01; *** *p* < 0.001.

To determine the cytotoxicity of *L. plantarum* K8 lysates and LPS against THP-1 cells, cells were treated with various concentrations of *L. plantarum* K8 lysates (10^6^, 10^7^, 10^8^, 10^9^ colony forming unit (CFU)/mL; 18 h) and/or LPS (31.25, 62.5, 125, 250, 500, 1000 ng/mL; 4 h). LPS did not induce a significant change of cell viability (Figure 1A). The treatment with only *L. plantarum* K8 lysates or LPS treatment following K8 lysate treatment affected cell survival at a concentration of 10^9^ CFU/mL of K8 lysates by 7.24 and 7.75%, respectively, but this was not significant (Figure 1B).

### 2.2. L. plantarum K8 Lysates Inhibited LPS-Induced TNF-α Production

THP-1 cells were stimulated with *L. plantarum* K8 lysates and/or LPS and the production of TNF-α was examined. The production of TNF-α increased in response to LPS in a dose-dependent manner (Figure 1C). We found that 10^8^ CFU/mL and 10^9^ CFU/mL of *L. plantarum* K8 lysates induced TNF-α, but the level of TNF-α was much lower than that observed in LPS-treated cells (Figure 1D). To examine the anti-inflammatory effect of the *L. plantarum* K8 lysates, THP-1 cells were pre-treated with *L. plantarum* K8 lysates for 18 h and then treated with LPS (500 ng/mL) for 4 h. LPS-induced TNF-α production in *L. plantarum* K8 lysate-pretreated THP-1 cells was reduced in a dose-dependent manner from 10^7^ to 10^8^ CFU/mL compared with THP-1 cells treated with LPS alone, but the level was significantly increased at 10^9^ CFU/mL. Treatment of cells with 10^7^, 10^8^, and 10^9^ CFU/mL *L. plantarum* K8 lysates reduced TNF-α levels by 22.46 ± 4.9, 78.67 ± 0.18, 35.55 ± 5.26%, respectively, compared with LPS-treated cells (Figure 1E). These results suggest that 10^8^ CFU/mL of *L. plantarum* K8 lysates may be effective for the prevention of pro-inflammatory diseases caused by pathogenic ligands such as LPS.

### 2.3. L. plantarum K8 Lysates Down-Regulated the LPS-Induced Signaling Pathways

**Figure 2:**
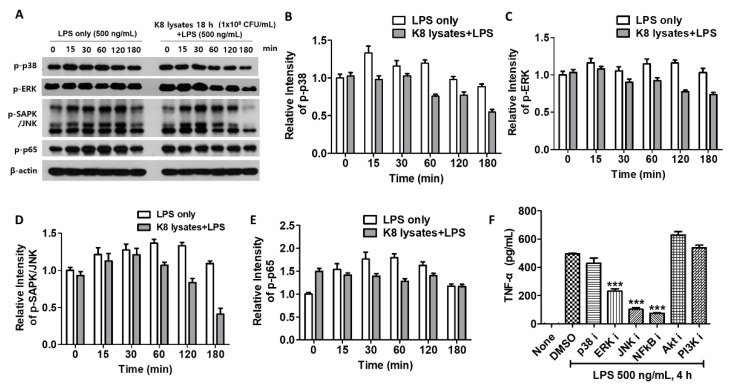
Down-regulation of signaling pathways by *L. plantarum* K8 lysates pretreatment: (**A**) THP-1 cells were pretreated with either medium or 1 × 10^8^ CFU/mL of *L. plantarum* K8 lysates for 18 h and then stimulated with 500 ng/mL LPS for the indicated time (min). The protein levels of p-p38, p-extracellular signal-regulated kinase (ERK), p-c-Jun N-terminal kinase (JNK), p-nuclear factor (NF)-κB, and β-actin in cell lysates were measured by western blot analysis. (**B**–**E**) The relative intensities of bands in (**A**) were measured by Image J software and shown. (**F**) THP-1 cells were pretreated with either (dimethyl sulfoxide) DMSO or 10 µM of inhibitors for 30 min and then treated with 500 ng/mL LPS for 4 h. The TNF-α level in the culture supernatants was determined using ELISA. Statistical analysis was conducted with one-way ANOVA followed by Tukey’s HSD post hoc test. *** *p* < 0.001.

In THP-1 cells, LPS-mediated phosphorylation of p38 peaked at 15 min and others were peaked at 60 min. However, upon pre-treatment with 10^8^ CFU/mL of *L. plantarum* K8 lysates, the phosphorylation of signaling molecules was decreased compared with controls. Phosphorylation of p38 and c-Jun N-terminal kinase (JNK) decreased at 60 min after LPS treatment, and extracellular signal-regulated kinase (ERK) and nuclear factor (NF)-κB decreased at 30 min after LPS treatment (Figure 2A). Quantification of the western blot results is shown in Figure 2B–E. To further determine whether *L. plantarum* K8 lysates inhibit LPS-induced TNF-α production by blocking signaling pathways, THP-1 cells were incubated with 10 μM inhibitors of p38, ERK, JNK, NF-κB, serine/threonine-specific protein kinase (Akt), and Phosphoinositide 3-kinases (PI3K) before LPS stimulation, and the culture supernatant was used for TNF-α analysis. TNF-α production did not increase in cells pre-treated with inhibitors of ERK, JNK, and NF-κB, indicating that these pathways were related to LPS-induced TNF-α production (Figure 2F). These results suggest that *L. plantarum* K8 lysates appear to control LPS-induced TNF-α production by mildly inhibiting overall related signaling pathways.

### 2.4. L. plantarum K8 Lysates Up-Regulated the LPS-TLR4 Pathway by Regulating Negative Regulators

**Figure 3:**
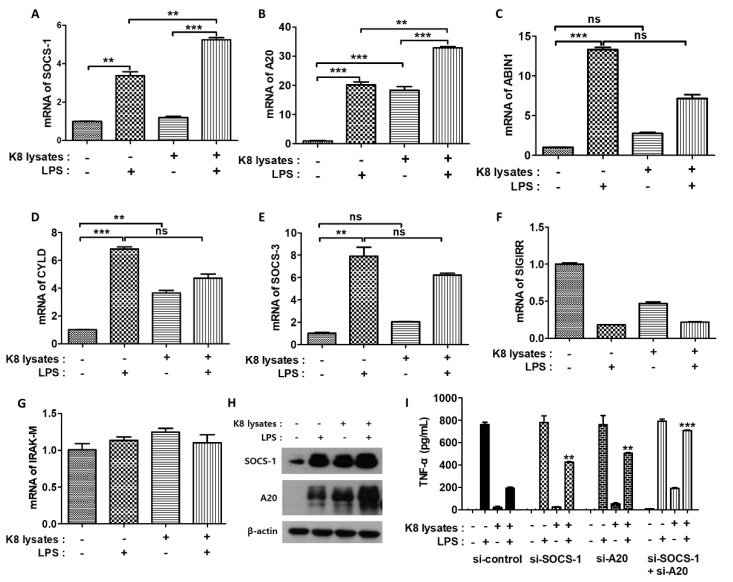
Suppressor of cytokine signaling (SOCS)-1 and tumor necrosis factor, alpha-induced protein 3 (A20) were involved in the down-regulation of signaling pathway initiated by *L. plantarum* K8 lysates and LPS. THP-1 cells were treated with 1 × 10^8^ CFU/mL *L. plantarum* K8 lysates for 18 h and/or 500 ng/mL of LPS for 4 h. mRNA levels of SOCS-1 (**A**), A20 (**B**), A20-binding inhibitor of NF-κB (ABIN1) (**C**), CYLD lysine 63 deubiquitinase (CYLD) (**D**), SOCS-3 (**E**), single Ig IL-1-related receptor (SIGIRR) (**F**), and interleukin-1 receptor-associated kinase (IRAK)-M (**G**) were measured by real-time PCR. (C) Protein levels of SOCS-1, A20 and β-actin were measured by western blot analysis. (**H**) THP-1 cells were transiently transfected with 25 nM of control (SI03650318), SOCS-1 (GS8651), or A20 (SG7128) siRNA purchased from Qiagen. After 36 h transfection, cells were stimulated with *L. plantarum* K8 lysates and/or LPS. The TNF-α level in the culture supernatants was determined using ELISA. Statistical analysis was conducted with one-way ANOVA followed by Tukey’s HSD post hoc test (**A**–**G**) and two-way ANOVA (**I**). ** *p* < 0.01; *** *p* < 0.001; ns, not significant.

LPS induces inflammatory cytokines primarily through the TLR4 pathway [25]. We next examined the mRNA expression of negative regulators associated with LPS-TLR4 signaling. Suppressor of cytokine signaling (SOCS)-1 mRNA increased 3.3-fold and tumor necrosis factor, alpha-induced protein 3 (A20) mRNA increased 20-fold in LPS-treated cells compared with controls. In cells treated with only *L. plantarum* K8 lysates, SOCS-1 mRNA did not increase but A20 mRNA increased 18-fold. Moreover, both SOCS-1 and A20 mRNA showed the greatest increase (fivefold and 32-fold) when THP-1 cells were co-treated with 10^8^ CFU/mL *L. plantarum* K8 lysates and LPS (Figure 3A,B). The mRNA levels of other negative regulators of LPS-TLR4 signaling such as A20-binding inhibitor of NF-κB (ABIN1), CYLD lysine 63 deubiquitinase (CYLD), SOCS-3, single Ig IL-1-related receptor (SIGIRR), and interleukin-1 receptor-associated kinase (IRAK)-M were unchanged in response to *L. plantarum* K8 lysates and LPS (Figure 3C–G). We further examined the protein levels of the negative regulators by western blot. Similar to mRNA expressions, SOCS-1 and A20 proteins were the most induced in *L. plantarum* K8 lysate-pretreated and LPS-treated cells (Figure 3H). To confirm that pretreatment with *L. plantarum* K8 lysates increased the expression of SOCS-1 and A20, thereby inhibiting LPS-induced TNF-α production, we used siRNA. THP-1 cells were transfected with 25 nM siRNA for 36 h and then treated with 10^8^ CFU/mL of *L. plantarum* K8 lysates and/or LPS. In the control siRNA groups, TNF-α in *L. plantarum* K8 lysate-treated cells decreased by 74.73% compared with levels in LPS-treated cells. In contrast, TNF-α decreased by 45.42% and 33.74% in *L. plantarum* K8 lysate-treated cells compared with LPS-treated cells in the si-SOCS-1 and si-A20-treated groups, respectively. In cells transfected with both si-SOCS-1 and si-A20, we observed a further reduction (10.6%) of TNF-α (Figure 3I). These results indicate that 10^8^ CFU/mL of *L. plantarum* K8 lysates increased the mRNA and protein levels of TLR4-related negative regulators such as SOCS-1 and A20, thereby inhibiting LPS-induced TNF-α production in THP-1 cells.

### 2.5. L. plantarum K8 Lysates Induced IL-1β Production

**Figure 4:**
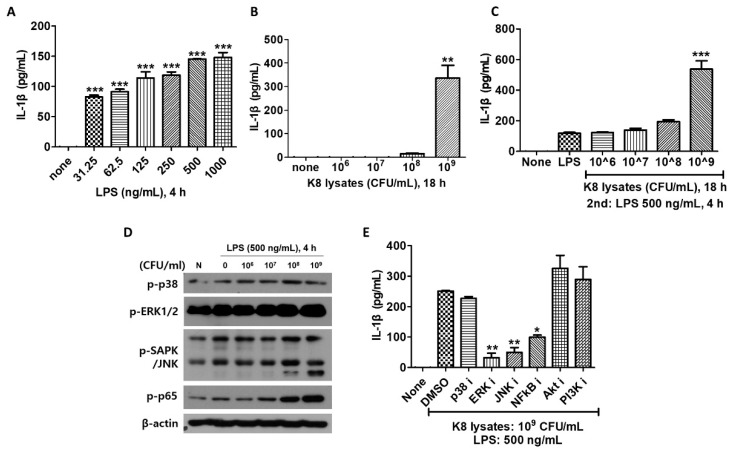
The production of IL-1β was increased in THP-1 cells by *L. plantarum* K8 lysates and LPS combination treatment. (**A**) THP-1 cells were treated with LPS at the indicated concentrations for 4 h. (**B**) THP-1 cells were treated with *L. plantarum* K8 lysates from 1 × 10^6^ to 1 × 10^9^ CFU/mL for 18 h. (**C**) THP-1 cells were pretreated with *L. plantarum* K8 lysates at the indicated concentrations for 18 h and treated with 500 ng/mL LPS for 4 h. In (**A**–**C**), the IL-1β level in the culture supernatants was determined using ELISA. (**D**) THP-1 cells were pretreated with indicated dose of *L. plantarum* K8 lysates for 18 h and then stimulated with 500 ng/mL LPS for 4 h. The protein levels of p-p38, p-ERK, p-SAPK/JNK, p-NF-κB p65, and β-actin were measured in cell lysates by western blot. (**E**) THP-1 cells were pretreated with either DMSO or 10 µM of inhibitors for 30 min. The cells were treated with 1 × 10^9^ CFU/mL of *L. plantarum* K8 lysates for 18 h and 500 ng/mL LPS for 4 h. The IL-1β level in the culture supernatants was determined using ELISA. Statistical analysis was conducted with one-way ANOVA followed by Tukey’s HSD post hoc test (**A**–**E**) and two-way ANOVA (G). * *p* < 0.05; ** *p* < 0.01; *** *p* < 0.001.

We next examined IL-1β production in THP-1 cells after cells were stimulated with *L. plantarum* K8 lysates and/or LPS. IL-1β increased in response to LPS in a dose-dependent manner (Figure 4A). In experiments on cells treated with 10^6^ to 10^9^ CFU/mL of *L. plantarum* K8 lysates, only 10^9^ CFU/mL of K8 lysates induced IL-1β (Figure 4B). IL-1β production in *L. plantarum* K8 lysates-treated and LPS-treated THP-1 cells was increased to higher levels than cells with LPS or *L. plantarum* K8 lysate only treatment. IL-1β increased with increasing concentration of *L. plantarum* K8 lysates, especially at 10^9^ CFU/mL. In cells treated with 10^6^, 10^7^, 10^8^, and 10^9^ CFU/mL K8 lysates, IL-1β increased by 1.03, 1.18, 1.63 and 4.51 times, respectively, compared with cells treated with only LPS (Figure 4C). This effect is noticeably different than our previous results showing a reduction of TNF-α in the *L. plantarum* K8 lysates-pretreatment group compared with the LPS treatment group. These results suggest that 10^9^ CFU/mL of *L. plantarum* K8 lysates may be an effective substance to increase immunity by inducing the inflammatory cytokine IL-1β. As the pretreatment with *L. plantarum* K8 lysates increased, LPS-induced phosphorylation of ERK, JNK and NF-κB increased by late signaling in THP-1 cells. The increase in phosphorylation was particularly significant in cells pretreated with 10^8^ and 10^9^ CFU/mL K8 lysates (Figure 4D).

To evaluate whether *L. plantarum* K8 lysates activate specific signaling pathways to induce IL-1β, cells were treated with 10 µM inhibitors and then stimulated with *L. plantarum* K8 lysates and LPS. The culture supernatant was used for IL-1β analysis. IL-1β did not increase in cells treated with ERK, JNK and NF-κB inhibitors (Figure 4E). Together, these results indicate that the *L. plantarum* K8 lysates increase IL-1β production by activating the late signaling of ERK, JNK and NF-κB.

### 2.6. L. plantarum K8 Lysates Recruited the Absent in Melanoma 2 (AIM2) Inflammasome and Secreted Active Mature IL-1β

**Figure 5:**
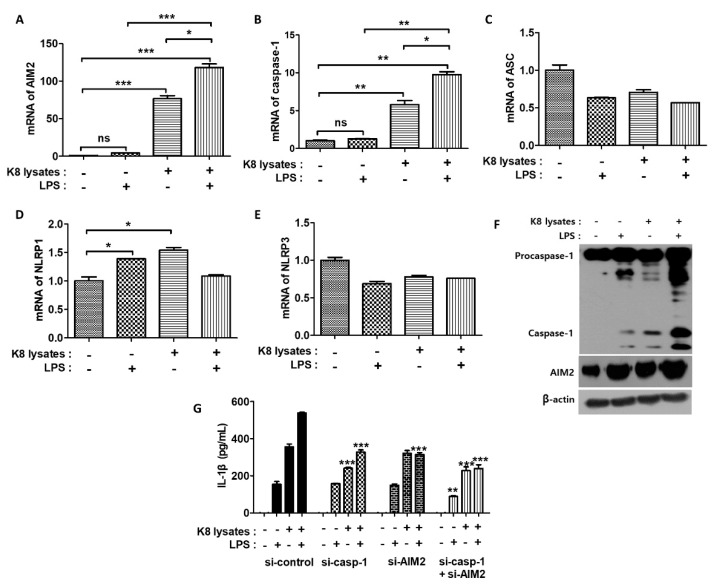
The regulation of inflammasomes by *L. plantarum* K8 lysates and LPS treatment. THP-1 cells were treated with 1 × 10^9^ CFU/mL of *L. plantarum* K8 lysates for 18 h and/or 500 ng/mL of LPS for 4 h. mRNA levels of absent in melanoma 2 (AIM2) (**A**), caspase-1 (**B**), apoptosis-associated Speck-like protein containing a CARD (ASC) (**C**), NLR family pyrin domain containing (NLRP) 1 (**D**), and NLRP3 (**E**) were measured by real-time PCR. (**F**) Protein levels of AIM2, caspase-1, and β-actin were measured by western blot. (**G**) THP-1 cells were transiently transfected with control siRNA, AIM2 siRNA (GS9447), and caspase-1 siRNA (GS834) purchased from Qiagen. After 36 h transfection, cells were stimulated with *L. plantarum* K8 lysates and/or LPS. The IL-1β level in the culture supernatants was determined using ELISA. Statistical analysis was conducted with one-way ANOVA followed by Tukey’s HSD post hoc test. * *p* < 0.05; ** *p* < 0.01; *** *p* < 0.001.

The AIM2 inflammasome is a detector of cytosolic double-stranded DNA (dsDNA) and plays an important role in the coordination of the immune defense against intracellular bacterial infections [26]. AIM2 forms pro-caspase-1-containing inflammasomes to cause pyroptosis or activate the maturation of the pro-inflammatory cytokine IL-1β [26]. We next investigated the mRNA expressions of inflammasome-related genes. Pre-treatment of *L. plantarum* K8 lysates and re-treatment of LPS increased the mRNA expression of AIM2 and caspase-1 in THP-1 cells. AIM2 mRNA levels increased by 4.4, 76.5, and 118.2 times in response to LPS, *L. plantarum* K8 lysates, and the combination treatment of *L. plantarum* K8 lysates and LPS, respectively, compared with untreated cells (Figure 5A). Caspase-1 mRNA levels increased by 1.3, 5.8 and 9.8 times, respectively (Figure 5B). In contrast, the mRNA expression of apoptosis-associated Speck-like protein containing a CARD (ASC), NLR family pyrin domain containing (NLRP) 1, and NLRP3 did not show any significant changes in response to *L. plantarum* K8 lysates and LPS (Figure 5C–E). We also examined the protein levels of AIM2 and caspase-1 by western blot. Similar to the mRNA expressions, both proteins were induced at the highest levels in response to the combination treatment of *L. plantarum* K8 lysates and LPS (Figure 5F).

To determine whether treatment with *L. plantarum* K8 lysates and LPS increased the expression of AIM2 and caspase-1 to induce IL-1β maturation, cells were transiently transfected with siRNA to knock-down AIM2 and caspase-1 genes. IL-1β secretion in cells treated with *L. plantarum* K8 lysates followed by LPS decreased by 41.7% and 39.1% in si-AIM2 and si-caspase-1 groups, respectively, compared with the siRNA-control group. When si-AIM2 and si-caspase-1 RNAs were co-transfected, IL-1β secretion was reduced by 55.6% compared with the siRNA-control group (Figure 5G). These results indicate that pre-treatment of 10^9^ CFU/mL *L. plantarum* K8 lysates and treatment of LPS increased the formation of the AIM2 inflammasome, which resulted in the induction of IL-1β maturation and secretion in THP-1 cells.

### 2.7. L. plantarum K8 Lysates Suppressed LPS-Induced Endotoxin Shock in Mice

**Figure 6:**
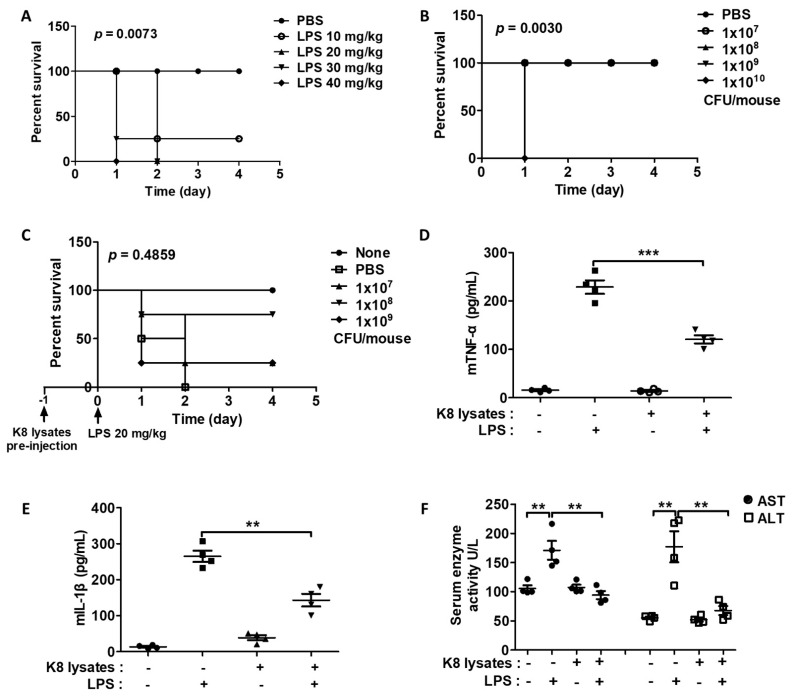
Co-treatment of *L. plantarum* K8 lysates and LPS decreased the mortality of endotoxin shock mice (**A**) BALB/C mice (seven-weeks-old, n = 4) were intraperitoneal (IP) injected with LPS from 10 mg/kg to 40 mg/kg. (**B**) Mice (n = 4) were IP injected with *L. plantarum* K8 lysates at the indicated concentrations. (**C**) Mice (n = 4) were IP pre-injected with PBS or indicated concentrations of *L. plantarum* K8 lysates; after 24 h, mice were IP injected with 20 mg/kg of LPS. In all experiments, the survival rate was monitored every 12 h. *p* values were derived by the log-rank (Mantel–Cox) test using GraphPad Prism 5 software (**A**–**C**). Mouse serum was collected 8 h after IP injection of 20 mg/kg LPS at 24 h after IP injection of PBS or 1 × 10^8^ CFU/mouse of *L. plantarum* K8 lysates. The TNF-α (**D**) and IL-1β (**E**) levels in serum were determined by ELISA. (**F**) The effect of *L. plantarum* K8 lysates on serum aspartate aminotransferase (AST) and alanine aminotransferase (ALT) was observed. Statistical analyses were conducted with an unpaired two-tailed *t*-test (**D**–**F**). ** *p* < 0.01; *** *p* < 0.001.

We next examined the effects of *L. plantarum* K8 lysates on endotoxin shock in BALB/c mice induced by LPS by monitoring the survival rate of mice. Among mice that received IP injection of various concentrations of an aqueous solution containing LPS, all mice injected with 20 to 40 mg/kg LPS died within 48 h, but 10 mg/kg LPS-injected mice did not die (Figure 6A). Among mice that received *L. plantarum* K8 lysates (10^7^, 10^8^, 10^9^, 10^10^ CFU/mouse), mice injected with 10^7^ to 10^9^ CFU/mouse *L. plantarum* K8 lysates survived, while the group injected with 10^10^ CFU/mouse *L. plantarum* K8 lysates died within 24 h (Figure 6B). Interestingly, IP pre-injection of 10^7^, 10^8^, and 10^9^ CFU/mouse *L. plantarum* K8 lysates in mice 24 h before injection of 20 mg/kg LPS improved survival rates by 25%, 75%, and 25%, respectively, compared with mice that only received LPS injection (Figure 6C). In addition, the group subjected to IP pre-injection of 10^8^ CFU/mouse K8 lysates showed decreased serum TNF-α by 50% and IL-1β by 55% compared with the mice only injected with LPS (Figure 6D and 6E). The LPS group had higher serum aspartate aminotransferase (AST) and alanine aminotransferase (ALT) levels than none. However, pre-injection of 10^8^ CFU/mouse *L. plantarum* K8 lysates reduced AST by 44% and ALT by 74% (Figure 6F). Mice injected with only *L. plantarum* K8 lysates showed no significant changes in cytokines, AST, and ALT levels compared to none.

### 2.8. L. plantarum K8 Lysates Suppressed LPS-Induced Liver Damage

**Figure 7:**
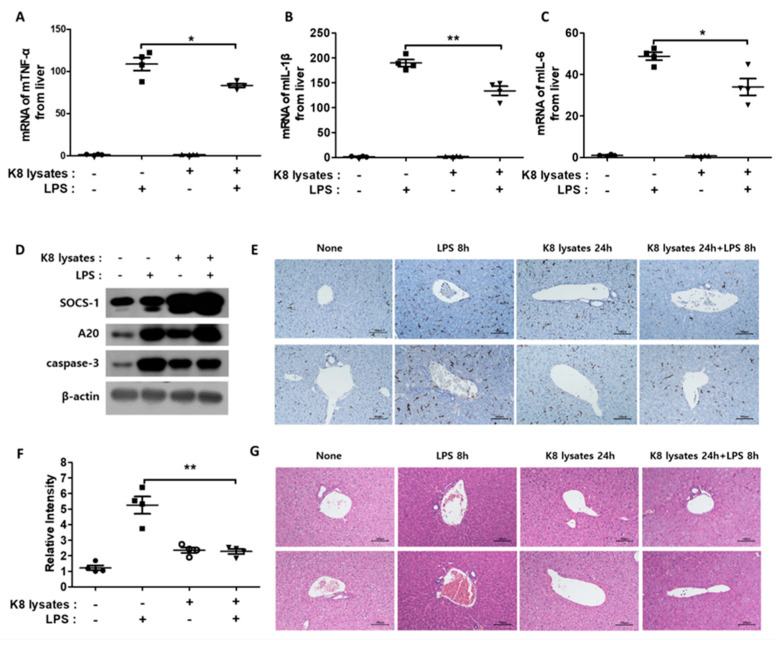
Co-treatment of *L. plantarum* K8 lysates and LPS reduced liver damage in mice: BALB/C mice (seven-weeks-old, n = 4) were IP injected with 20 mg/kg LPS after IP pre-injection of PBS or 1 × 10^8^ CFU/mouse of *L. plantarum* K8 lysates for 24 h. mRNA and protein were extracted from liver homogenates. The mRNA expressions of TNF-α (**A**) and IL-1β (**B**), and IL-6 (**C**) were determined by real-time PCR. (**D**) Protein levels of SOCS-1, A20, caspase-3, and β-actin in liver were measured by western blot analysis. (**E**) Immunohistochemistry of F4/80 in livers and (**F**) relative intensity of F4/80. (**G**) Hematoxylin and eosin (H&E) staining of liver from the indicated mice. In (**E**,**G**), two representative liver images out of four liver samples are shown. Statistical analyses were conducted with an unpaired two-tailed *t*-test. * *p* < 0.05; ** *p* < 0.01.

A previous study reported that the intraperitoneal (IP) injection of LPS causes liver damage [27]. Thus, we examined whether *L. plantarum* K8 lysates inhibited LPS-induced liver damage. LPS injection triggered an increase in TNF-α, IL-1β, and IL-6 in the liver. However, pre-injection of *L. plantarum* K8 lysates lowered the expression of these cytokines (Figure 7A–C). In addition, similar to the results of in vitro experiments, pre-injection of *L. plantarum* K8 lysates and re-injection of LPS increased the protein expression of the negative regulators SOCS-1 and A20 (Figure 7D). Liver damage is accompanied by hepatocyte cell death, which occurs with an increase in activated caspase protein [28]. Hepatic caspase-3 was significantly increased in LPS-injected mice, but pre-injection of *L. plantarum* K8 lysates reduced caspase-3 expression. The level of caspase-3 was similar to that of the *L. plantarum* K8 lysate injection without LPS (Figure 7D). If the liver is damaged, macrophage infiltration also increases [29]. To evaluate macrophage infiltration, immunohistochemistry (IHC) with an antibody for the mouse macrophage receptor F4/80 was performed. The black part in Figure 7E represents F4/80, and Figure 7F represents the relative intensity of F4/80 area in Figure 7E. A 50% reduction of macrophage infiltration was observed in mice pre-injected with *L. plantarum* K8 lysates compared with mice that only received LPS injection. In the additional study, hepatocyte swelling was observed when LPS was injected. However, in mice with *L. plantarum* K8 lysates pre-injection, the cells were less swollen compared with the LPS group (Figure 7G). Taken together, these results suggest that *L. plantarum* K8 lysates reduced the lethality of endotoxin shock in mice by inhibiting inflammatory responses.

## 3. Discussion

The human body triggers an inflammatory reaction, protecting it from external stimuli or the ingress of harmful substances. However, excessive inflammatory reactions can lead to serious problems such as septic shock and autoimmune diseases [30]. Therefore, substantial research has investigated substances that inhibit excessive inflammation. Probiotics represent a good option for addressing inflammation. For example, *Lactiplantibacillus plantarum (L. plantarum)*, widely known to have immune-boosting and anti-inflammatory effects, is commonly consumed as a dietary supplement. *L. plantarum* is mainly distributed as live bacteria, and much effort is being made to maintain the number of live bacteria during the distribution process [31]. Some *L. plantarum* strains have high probiotic potential by regulating inflammatory responses. For example, *L. plantarum* A41 strain increases gut barrier function by upregulating the expression of tight junction-related genes and inhibiting the expression of inflammatory mediators induced by LPS stimulation. This strain can also modulate several markers involved in bone metabolism, which play a role in bone homeostasis and osteoblast differentiation [16]. *L. plantarum* KU15149 strain has high antioxidant activity, which inhibits LPS-mediated nitric oxide (NO) production, cyclooxygenase-2 (COX-2), and proinflammatory cytokines such as TNF-α, IL-1β, and IL-6 in RAW 264.7 cells [17]. In addition to live bacteria, heat-killed *L. plantarum* also shows strong anti-inflammatory effects. Heat-killed *L. plantarum* L-137 strain attenuates left ventricular inflammation and fibrosis as well as adipocyte hypertrophy and inflammation in DS/obese rats, suggesting that heat-killed *L. plantarum* L-137 has beneficial effects on the heart and adipose tissues [32]. To avoid the potential risks of live probiotics including systemic infections due to translocation, the acquisition of antibiotic resistance genes or interference with gut colonization in neonates, heat-killed probiotic bacteria and bacterial extracts were considered as an alternative probiotic [33]. The production lines using heat-killed bacteria or bacterial lysates are being released in the industry. Probiotic culture supernatants of some species of probiotics such as *L. acidophilus, L. casei, L. lactis, L. reuteri*, and *Saccharomyces boulardii* inhibited the expression of TNF-α, IL-1β, IL-6, and IL-10 from macrophages, suggesting that metabolites produced by probiotics exert their anti-inflammatory effects [34]. Although specific strain of probiotics such as *L. plantarum* induces anti-inflammatory effects, it can also induce strong anti-inflammatory effects through multiple probiotic formulas [35].

Previous studies examined the anti-inflammatory efficacy of LTA isolated from the *L. plantarum* K8 cell wall (pLTA). pLTA inhibited both the expression of inflammatory cytokines such as TNF-α, IL-1β, and IL-6 and activation of the MAPK and NF-κB pathways [21]. However, *L. plantarum* K8 lysates showed different results depending on the cytokine. Even with the same concentration of lysates, LPS-induced TNF-α induction was suppressed, whereas IL-1β increased. Unlike pLTA, inflammasome seems to be involved in the increase of IL-1β expression in the cells treated with *L. plantarum* K8 lysates. Although it has not yet been examined whether pLTA affects the formation of inflammasomes, *L. plantarum* K8 lysates certainly seem to induce the formation of inflammasomes. Through experiments, we confirmed that *L. plantarum* K8 lysates increased the production of active caspase-1 through combination with LPS. Caspase-1, along with ASC or AIM2 and NLRP, is an important key element in the formation of inflammasomes. Activated caspase-1 is involved in the maturation of IL-1β [36,37,38]. We also observed differences in the activation of signaling molecules induced by pLTA and *L. plantarum* K8 lysates. While pLTA inhibited both NF-κB and MAPKs activated by LPS within 2 h [21], *L. plantarum* K8 lysate did not significantly inhibit the activity of certain signaling substances by LPS within 2 h. However, combination treatment of *L. plantarum* K8 lysates and LPS induced the activation of NF-κB and JNK after 4 h treatment, which was associated with the induction of IL-1β expression (Figure 4).

In the combination treatment of *L. plantarum* K8 lysates and LPS, we found that 10^8^ CFU/mL *L. plantarum* K8 lysates inhibited the expression of TNF-α induced by LPS, but 10^9^ CFU/mL *L. plantarum* K8 lysates increased it. This phenomenon was repeatedly observed in various experiments using *L. plantarum* K8 lysates. We consider this phenomenon as one of the immunoregulatory phenomena of *L. plantarum* K8 lysates. *L. plantarum* K8 lysates suppressed excessive inflammatory reactions by regulating the expression of TNF-α and might maintain immune homeostasis by inducing an increase in the expression of IL-1β as an immediate countermeasure. The opposite expression pattern of TNF-α and IL-1β may be the result of the negative regulators SOCS-1 and A20. They selectively inhibited the TNF-α expression pathway, while not blocking the IL-1β pathway (Figure 3). A similar phenomenon can be found in our previous paper. THP-1 cells regulate the expression of IL-6 in response to LPS, and the expression of IL-6 decreases or increases depending on the concentration of LTA [39].

Similar and consistent results were obtained with pLTA and *L. plantarum* K8 lysates in in vivo results. When LPS was IP injected 24 h after IP injection of *L. plantarum* K8 lysates, TNF-α, IL-1β, AST and ALT in serum were all decreased compared with mice that only received LPS injection. In addition, the mRNA levels of TNF-α, IL-1β, and IL-6 in liver homogenates were decreased in mice injected with *L. plantarum* K8 lysates and LPS. This is quite different from the in vitro experiments in which TNF-α decreased and IL-1β increased. Several types of immune cells such as monocytes, lymphocytes, neutrophils, eosinophils, basophils, and macrophages are present in blood. These cells produce different types of cytokines in response to the same stimulus [40]. This may be the reason that the in vivo results are different from the in vitro results performed with THP-1 cells.

Together, these results indicate that *L. plantarum* K8 lysates reduce inflammation by reducing inflammatory cytokines induced by bacterial toxins such as LPS. *L. plantarum* K8 lysates, which represent a new material with an anti-inflammatory effect, with the possibility for development for industrial use.

## 4. Materials and Methods

### 4.1. Cell Culture

THP-1 cells, a human monocyte-like cell line, were maintained in RPMI-1640 medium supplemented with 10% heat-inactivated fetal bovine serum (FBS), 100 μg/mL penicillin, and 100 μg/mL streptomycin at 37 °C in a humidified 5% CO_2_ incubator. The cells were sub-cultured at every three to four days. For experiments, THP-1 cells were seeded onto 96- or six-well plates; after incubation for 24 h, cells were stimulated with *L. plantarum* K8 lysates and/or LPS (*Escherichia coli* 055:B5; Sigma-Aldrich, St. Louis, MO, USA).

### 4.2. Preparation and Modification of L. plantarum K8 Lysates

*L. plantarum* K8 was cultured in 1 L of MRS broth (BD Bioscience, San Jose, CA, USA) at 37 °C overnight and then cells were harvested by centrifugation at 8000× *g* rpm for 8 min. Cells were re-suspended in sterilized water and disrupted by a microfluidizer five times at 27,000 psi. Disrupted *L. plantarum* K8 was freeze-dried and re-suspended in PBS at a concentration of 10^11^ colony-forming units (CFUs)/mL for subsequent experiments.

### 4.3. Cell Viability Test (Trypan Blue Assay)

THP-1 cells were seeded onto 96-well plates and incubated overnight. The cells were stimulated with *L. plantarum* K8 lysates (10^6^, 10^7^, 10^8^, 10^9^ CFU/mL) for 18 h and/or LPS (31.25, 62.5, 125, 250, 500, 1000 ng/mL) for 4 h. Next, 0.4% Trypan blue solution (Sigma-Aldrich) was added to the cells. Cell viability was determined by dividing the number of viable cells (unstained) by the number of total cells.

### 4.4. Enzyme-Linked Immunosorbent Assay (ELISA)

After the cells were stimulated with *L. plantarum* K8 lysates and/or LPS, culture supernatants were collected and used for TNF-α ELISA assays. The human TNF-α capture antibody (#MAB610), human TNF-α biotinylated antibody (#BAF210), human IL-1β/IL-1F2 DuoSet ELISA (#DY201-05), mouse TNF-α capture antibody (#AF-410-NA), mouse TNF-α biotinylated antibody (#BAF410), mouse IL-1β capture antibody (#MAB401), and mouse IL-1β biotinylated antibody (#BAF401) were used according to the manufacturer’s instructions (R&D Systems, Minneapolis, MN, USA).

### 4.5. Real-Time PCR

Total RNA was extracted from THP-1 cells with RNAiso PLUS reagent (Takara, Shiga, Japan) and cDNA was synthesized using Prime Script^TM^ RT Master Mix (Takara). To quantify mRNA expression, real-time PCR amplification was conducted using the CFX Connect^TM^ real-time PCR detection system (Bio-Rad, Hercules, CA, USA) and the PCR products were detected with TB Green^TM^ Premix EX Taq^TM^ II (Takara). The forward and reverse primer pairs were listed in Supplementary Table S1. The expression of mRNA was normalized using human or mouse glyceraldehyde 3-phosphate dehydrogenase (GAPDH) mRNA.

### 4.6. Western Blot Analysis

THP-1 cells stimulated with *L. plantarum* K8 lysates and/or LPS were harvested and centrifuged for 10 min at 12,000× *g* rpm. Pellets were lysed with pro-prep cell lysis buffer (iNtRON Biotechnolog, Seongnam, Korea) and the protein concentration was quantified by Bradford assay. Samples were then mixed with 2x Laemmli buffer and boiled for 5 min. Protein samples were resolved by 10% or 12% SDS-PAGE in a Tris/glycine/SDS buffer (25 mM Tris, 250 mM glycine, 0.1% SDS) and transferred onto PVDF membranes (EMD Millipore, MA, USA) overnight (40 V, 4 °C). The membranes were blocked for 2 h with TBST (20 mM Tris-HCL, 150 mM NaCl, 0.1% Tween 20) containing 5% BSA and then washed three times with TBST. The membranes were then incubated with primary antibodies such as anti-phospho-p38 (#9211), anti-phospho-p44/42 (#9101), anti-phospho-SAPK/JNK (#9251), anti-phospho-NFκBp65 (#3033), anti-A20 (#5630), anti-AIM2 (#12948), anti-caspase-1 (#2225), anti-caspase-3 (#9662) antibodies (Cell Signaling Technology, Danvers, MA, USA), anti-SOCS1 (sc-518028), and anti-β-actin HRP (sc-47778) (Santa Cruz Biotechnology, Dallas, CA, USA) for 2 h at RT. After washing the membrane three times with TBST, HRP-conjugated anti-rabbit (sc-2357, Santa Cruz Biotechnology) or anti-mouse (#31430, Invitrogen, Waltham, CA, USA) secondary antibodies were incubated with membranes for 2 h at RT and then the membranes were washed four times in TBST. Bands were detected using ECL reagents (Thermo Fisher Scientific, Waltham, MA, USA) and the blot was exposed to X-ray film.

### 4.7. In Vivo Study

Male BALB/c mice (seven-weeks-old, *n* = 4/group, Narabiotech, Seoul, Korea) were used for experiments. Endotoxin shock was induced by IP injection of LPS in PBS. In some experiments, mice were injected with various concentrations of LPS (10 to 40 mg/kg). In other experiments, mice were first IP injected with *L. plantarum* K8 lysates (1 × 10^7^ to 1 × 10^10^ CFU/mL) for 24 h, followed by injection of LPS (20 mg/kg). The survival rate of mice was monitored over four days with 12 h intervals. The TNF-α and IL-1β levels in serum were measured by ELISA. Aspartate aminotransferase (AST) and alanine aminotransferase (ALT) levels in serum were assayed using colorimetric assay kits (Biovision, Milpitas, CA, USA). The liver was collected for histological analysis and immunohistochemistry. Liver homogenates were used for real-time PCR of TNF-α, IL-1β, and IL-6 mRNA and western blot analysis of SOCS-1, A20 and caspase-3. This study was approved by the Institutional Animal Care and Use Committee of Kyung Hee University (KHUASP (GC)-18-033, 2018).

### 4.8. Histological Examinatinos

Liver sections were perfused with PBS for 10 min and fixed in 4% paraformaldehyde for 5 min. The isolated livers were embedded in Tissue-Tek OCT compound and frozen at −70 °C. All samples were sectioned using a cryostat at −20 °C, and six consecutive 5 μm thick sections were cut from the livers. For immunohistochemistry, sections were stained with fist anti-F4/80 (1/100 dilution, Rat IgG2b, clone CI:A3-1, Abcam) and secondary mouse monoclonal Alexa Fluor^®^ 488 antibody (1/250 dilution). Alternatively, liver sections were stained with Oil red O and counterstained with hematoxylin. To visualize the targets in the lesion, fluorescence-labeled antibodies were used and observed at an excitation of 488 nm.

### 4.9. Statistical Analysis

All experiments were performed at least three times. The data shown are representative results of the means ± standard deviation. Statistical analyses were conducted with an unpaired two-tailed *t*-test, one-way analysis of variance (ANOVA) followed by Tukey’s honestly significant difference (HSD) post hoc test, two-way ANOVA, or log-rank (Mantel–Cox) test. Differences were considered statically significant when the *p*-value was < 0.05. GraphPad Prism 5 software was used for the analysis.

## Data Availability

The data presented in this study are available on request from the corresponding authors.

## Supplementary Materials

The following are available online at https://www.mdpi.com/article/10.3390/ijms22115921/s1. Table S1: Primer sets used in the study.
