# Supplementary material for: Alleviation of LPS-Induced Inflammation and Septic Shock by Lactiplantibacillus plantarum K8 Lysates
Source: Int J Mol Sci. 2021 May 31;22(11):5921. doi: 10.3390/ijms22115921 (PMC8197946; doi:10.3390/ijms22115921)
Supplement: Supplementary file 1 [file ijms-22-05921-s001.zip › ijms-1208119-supplementary.pdf]

Supplement Table S1. Primer sets used in the study.

Gene	Primer	Sequence (5'→3')
hGAPDH	Forward	GTCTTCACCACCATGGAGAA
	Reverse	AGGAGGCATTGCTGATGAT
mGAPDH	Forward	TGCTGACAATCTTGAGTGAG
	Reverse	GTCGTGGAGTCTACTGGTGT
mTNF-α	Forward	ATGGCCTCCCTCTCATCAGT
	Reverse	TTTGCTACGACGTGGGCTAC
mIL-1β	Forward	CTAAAGTATGGGCTGGACTG
	Reverse	GGCTCTCTTTGAACAGAATG
mIL-6	Forward	CCCCAATTTCCAATGCTCTCC
	Reverse	GGATGGTCTTGGTCCTTAGCC
AIM2	Forward	TCCAGTTGTCACTCCTACCCA
	Reverse	ACACTTCTGGACGGCTTCAC

ASC	Forward	TTGGACCTCACCGACAAGC
	Reverse	ATGTCGCGCAGCACGTTA
NLRP1	Forward	GCATAGCCGTACCTTCACCT
	Reverse	ACAACCTCCACCGATGTCAC
NLRP3	Forward	GTGATCCTTCCGGTGGAGTG
	Reverse	CTCACCCTGGCGTAAAGGAG
SOCS-1	Forward	GTGCACGCAGCATTAACTGG
	Reverse	GGAGGGTACCCACATGGTTC
TNFAI3/A20	Forward	CAGTCTGCAGTCTTCGTGGC
	Reverse	GGTGTGATCTCTCTTGGCGG
ABIN-1	Forward	GGAGTTCAACCGACTGGCAT
	Reverse	CTTCCGAAGCTGCTCACACA
CYLD	Forward	ATGGTTCTACACAGCCACCC
	Reverse	TTTTCAGCAACGTGGTGTCC

SOCS-3	Forward	GGGGAGTACCACCTGAGTCT
	Reverse	TGTGGTTGCTATCGTCCCAC
SIGIRR	Forward	TTCTCCTCCTTCACTCTTCA
	Reverse	AGACGGCACTTGACATAGAG
IRAK-M	Forward	TTGGTCCTGGGCACAGAAAA
	Reverse	TCGAATGTGCCAAGGGAGTG
